# Perioperative and Long-Term Anatomical and Subjective Outcomes of Laparoscopic Pectopexy and Sacrospinous Ligament Suspension for POP-Q Stages II–IV Apical Prolapse

**DOI:** 10.3390/jcm11082215

**Published:** 2022-04-15

**Authors:** Paulina Szymczak, Magdalena Emilia Grzybowska, Sambor Sawicki, Konrad Futyma, Dariusz Grzegorz Wydra

**Affiliations:** 1 Department of Gynecology, Obstetrics and Neonatology, Medical University of Gdańsk, Smoluchowskiego 17, 80-214 Gdansk, Poland; paulina.szymczak89@gmail.com (P.S.); sambor.sawicki@gumed.edu.pl (S.S.); dwydra@icloud.com (D.G.W.); 2 2nd Department of Gynecology, Medical University of Lublin, Jaczewskiego 8, 20-954 Lublin, Poland; futymakonrad@mp.pl

**Keywords:** apical defect, laparoscopy, pectopexy, pelvic organ prolapse, sacrospinous ligament suspension

## Abstract

The aim of this paper was to analyze perioperative and long-term outcomes in 114 women undergoing surgery for POP-Q ≥ 2 apical prolapse: sacrospinous ligament colpo/hysteropexy (SSLF/SSHP)—61; laparoscopic pectopexy (LP)—53. Validated questionnaires (PGI-I, ISI, #35 EPIQ, PFIQ-7, PFDI-20) were completed at baseline and follow-up. POP-Q stages II, III and IV were diagnosed in 1 (0.9%), 84 (73.7%) and 29 (25.4%) patients, respectively. Mean operative time and hospital stay were 151.8 ± 36.2 min/2.6 ± 1.1 days for LP and 69 ± 20.4 min (*p* < 0.001)/2.7 ± 1.0 days for SSLF. Severe intraoperative complications occurred in two (1.8%) patients. Mean follow-up was 26.9 ± 12 and 37.3 ± 17.5 months for LP and SSLF, respectively. At follow-up, significant improvement for all POP-Q points was observed in both groups (*p* < 0.001). Shortening of total vaginal length was found in both groups, but predominantly in SSLF patients (*p* = 0.01). The sensation of vaginal bulge (EPIQ) was reduced, and total PFDI-20 and PFIQ-7 scores improved (*p* < 0.04) in both groups. Subjective success was reported by 40 (75.5%) LP and 44 (72.1%) SSLF patients. ISI detected no deterioration in urinary incontinence. PGI-I, PFDI-20, #35 EPIQ, PFIQ-7 and ISI did not differ between the groups. In conclusion both, SSLF and LP for apical prolapse generate good anatomical and subjective outcomes, with protective effect on the anterior compartment observed for LP.

## 1. Introduction

Some degree of pelvic organ prolapse (POP) is a common health issue for women, with prevalence estimated from 2.9–11.4%—when using questionnaires, to 31.8–97.7%—when using physical examination with the Pelvic Organ Prolapse Quantification System (POP-Q) [1,2]. Nowadays, minimally invasive techniques (laparoscopy or robotic-assisted laparoscopy), open abdominal procedures, and vaginal approaches remain the mainstay of POP surgery.

Apical defect is the least frequent of all POP types, with a prevalence of 0.5 to 15% [3,4]. Clinical manifestations of the loss of apical support include vaginal bulge or pressure and defecatory and sexual dysfunction [3]. Patients with POP have a high rate of coexisting pelvic floor disorders—40% will present with urinary incontinence (UI), 37% with overactive bladder (OAB), and 19.6% with solid stool, and 51.2% with liquid stool fecal incontinence (FI) [5,6]. These symptoms may have a major impact on the functional performance and quality of life (QoL) of the affected women, causing many limitations and alterations to their everyday functioning [3].

Transvaginal apical repair is mainly represented by the sacrospinous ligament suspension (SSLF) and uterosacral ligament suspension (USLS) [2,7]. Complications related to SSLF are widely known and include buttock pain, vascular injury and suture-related complications such as granulation tissue formation, bleeding, pain and discharge [8]. Pelvic hemorrhage from a laceration of the hypogastric venous plexus or pudendal vein remains the most severe intraoperative complication [9]. The SSLF procedure is also known as the Amreich–Richter procedure. Isidor Amreich and Kurt Richter were the first who performed apical vaginal fixation to the sacrotuberous ligament (1951) or sacrospinous ligament (1967), respectively [10].

Uterine suspension to the iliopectineal ligaments was first mentioned in 1993 by Joshi et al. [11]. In 2010, a report of 12 cases of laparoscopic approach pectopexy (LP) was described by Banerjee and Noe [12]. During this procedure, a polyvinylidene fluoride (PVDF) monofilament mesh is fixated to the iliopectineal ligaments and the vaginal apex or cervical stump, so the reliability of the cranial fixation can be consequently higher [13]. Possible complications of LP have not been studied to a great extent but may include typical laparoscopic complications (i.e., bowel, urinary bladder and ureter injuries; urinary retention; de novo UI; vascular injury) and mesh-related complications such as exposure or detachment [12,13,14,15,16,17,18]. Protective effect against de novo central and lateral cystocele has been reported as an important advantage of LP [13].

In light of the rapid development of various POP-related procedures and their modifications, it was necessary to standardize the terminology, which in turn improved the quality of the investigations and clinical care associated with these procedures [19]. According to the abovementioned report, SSLF is defined as the suspension of the vaginal apex to the unilateral or bilateral sacrospinous ligament(s) using suture. Nowadays, a significant group of women expect their uterus to be preserved during surgery. Apart from the anatomical success, protection of the uterus has been reported to improve sexual function, especially in young patients [20]. The advantages of preserving the uterus include maintenance of the pelvic anatomy, preservation of the reproductive function, reduction of intraoperative blood loss, shortened operative time and hospital stay, lower rates of mesh exposure as well as higher self-confidence and sexuality [20,21]. Additionally, in the absence of concomitant hysterectomy, which is a risk factor for POP in itself, future development of POP can be prevented [22]. In a recent study on LP, the authors concluded that preserving the uterus protects against de novo development of rectocele [20]. If the uterus or the uterine cervix are preserved, sacrospinous hysteropexy (SSHP) can be performed [19].

In the current report about terminology for surgical procedures to treat POP, iliopectineal suspension procedures are included in the anterior abdominal wall hysteropexy (AAWHP) group [19]. Pectopexy, as such, is not listed separately. Of late, the International Procedures Advisory Committee (IPAC) has examined laparoscopic pectopexy with mesh, and the National Institute for Health and Care Excellence (NICE) has issued a document for public consultation about its safety and efficacy [23].

Comparative data about SSLF and LP are limited. The present study compared the perioperative results and long-term anatomical and functional outcomes of LP and SSLF in a single university-based medical center.

## 2. Material and Methods

A prospective, observational study was based on the perioperative results of 114 women who had undergone SSLF or SSHP (*n* = 61) and LP (*n* = 53) due to symptomatic POP-Q ≥ 2 stage apical prolapse. Medical history was taken, and urogynecologic examinations were performed in accordance with the standards of the International Continence Society (ICS), including cough stress tests and perineal ultrasonography [24]. The ICS POP-Q examination was performed on maximal Valsalva maneuver in the lithotomy position for each patient independently by two authors. In our study, we used the commonly used POP-Q scale as follows:

Stage 0: No prolapse is demonstrated.
Stage I: The most distal portion of the prolapse is more than 1 cm above the level of the hymen.
Stage II: The most distal portion of the prolapse is situated between 1 cm above the hymen and 1 cm below the hymen.
Stage III: The most distal portion of the prolapse is more than 1 cm beyond the plane of the hymen but everted at least 2 cm less than the total vaginal length.
Stage IV: Complete eversion or eversion at least within 2 cm of the total length of the lower genital tract is demonstrated [24].

General patient characteristics, i.e., age, menopausal status, parity, POP-Q stage, previous POP surgeries and body mass index (BMI), were collected. Perioperative patient data, i.e., operative time, concurrent procedures, intraoperative complications, change in hemoglobin level and the length of hospital stay, were collected and analyzed. Follow-up data, i.e., change in pain intensity assessed with Visual Analogue Scale (VAS; score 0–10), anatomical cure rate (points C, Aa, Ba, Ap, Bp, TVL—total vaginal length), symptoms of de novo or persistent UI and fecal incontinence (FI), assessment of sexual activity, rehospitalizations and reoperations, were obtained at follow-up and analyzed. Operative time was estimated as time from the first skin incision to the final skin closure. The difference between pre- and post-operative hemoglobin levels was used to estimate blood loss. Time between surgery and discharge was employed to calculate the length of the hospital stay. Clavien–Dindo classification was applied to register surgical complications [25].

The follow-up visit took place between April and October 2021. Anatomical prolapse recurrence was defined in the apical compartment as the presence of point C ≥ −1 at the follow-up visit or apical defect retreatment (pessary or surgery). Stress urinary incontinence (SUI) was defined as urine leakage during a cough stress test. The diagnosis of urgency urinary incontinence (UUI) was based on the findings from a 3-day voiding diary [26].

**Figure 1:**
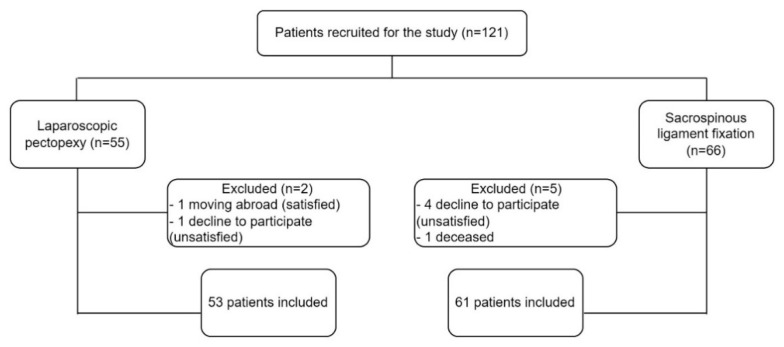
Study flowchart.

Patient subjective assessment of the treatment outcome is crucial when analyzing the functional outcomes of surgeries. All participants completed the Polish version of the Patient Global Impression of Improvement questionnaire for urogenital prolapse (PGI-I), pre- and postoperatively. PGI-I is a simple and feasible tool for determining the “success” following surgery and can be used in both clinical practice and in research settings. The PGI-I score indicates an overall improvement in patient perception of her post-treatment condition (PGI-I score 1–7, from very much better to very much worse) [27]. The Incontinence Severity Index (ISI), which is based on the information about frequency (four levels) and amount of leakage (two or three levels), was used pre- and postoperatively to evaluate urine loss. In the three-level severity index applied in our study, responses to the second question take the values (1) drops and (2) more, and are then multiplied with the frequency, resulting in the following index values (1–8): 1–2—slight; 3–4—moderate; and 6–8—severe [28]. The Pelvic Floor Impact Questionnaire short form (PFIQ-7) was used pre- and postoperatively to assess the degree to which bladder, bowel or vaginal symptoms affect daily activities, relationships, and emotions of women with POP. PFIQ consists of three separate domains, including the Urinary Impact Questionnaire (UIQ), the Colo-Rectal-Anal Impact Questionnaire (CRAIQ) and the Pelvic Organ Prolapse Impact Questionnaire (POPIQ) [29]. The Pelvic Floor Distress Inventory (PFDI-20) was yet another tool used in our study, pre- and postoperatively. PFDI-20 is a reliable, condition-specific questionnaire and also consists of three separate domains, including the Pelvic Organ Prolapse Distress Inventory (POPDI), the Colorectal–Anal Distress Inventory (CRADI) and the Urinary Distress Inventory (UDI) [30]. Question 35 from the Epidemiology of Prolapse and Incontinence Questionnaire (EPIQ; question 35) was used pre- and postoperatively to screen for symptoms of clinically significant apical defect [31]. At the follow-up visit, the patients were additionally asked three questions—“Are you satisfied with the surgery?”, “Did the surgery improve the symptoms you reported?” and “Would you recommend this surgery to a friend/colleague?”. Women with a history of apical prolapse surgery and those who did not answer the validated questionnaires at baseline, as well as lost to follow-up cases, were excluded from the study. The algorithm of patient recruitment is presented in Figure 1.

Our primary goal was to determine the anatomical success and patient-reported subjective outcomes after surgery. The secondary outcomes included the analysis of the intraoperative complications, rehospitalizations, reoperations and apical relapse.

### 2.1. Procedure Description

During SSLF, after injecting the saline solution under the posterior vaginal wall, a longitudinal incision was made in the midline, 3 cm distal to the vaginal apex or cervix. One canal in the right pararectal space, designated to admit the finger of the surgeon, was formed under the vaginal wall, horizontally in the direction of the pelvic side wall and, next, directly to the right sacrospinous ligament. Two nonabsorbable, polypropylene monofilament sutures were anchored to the right sacrospinous ligament using the Miya hook. The other end was sutured to the vaginal apex or posterior aspect of the cervical stroma. The vaginal incision was closed with a pre-positioned running suture.

LP was performed following the method introduced by Noé et al. [13]. Polypropylene mesh sized 20/35 × 159 mm was attached to the both iliopectineal ligaments (with one non-absorbable, braided suture) and fixed to the vaginal cuff or cervix (with 3–4, 0 polypropylene monofilament or braided polyester non-absorbable sutures). Finally, the mesh was covered with peritoneum using continuous absorbable suture in an endoscopic suturing technique.

**Figure 2:**
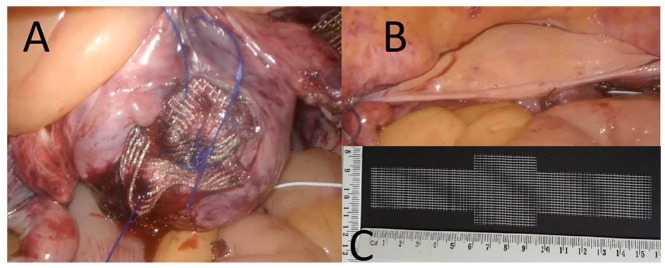
Laparoscopic pectopexy—(**A**) placement of mesh sutured to the vaginal vault, (**B**) final view from the end of the surgery and (**C**) mesh used in this procedure.

All patients have given written informed consent before surgery. The concomitant procedures were performed at the discretion of the surgeon and due to specific medical indications (Table 1). The final surgery effect of LP and the mesh used for this procedure are shown in Figure 2.

### 2.2. Statistical Analysis

Categorical variables were expressed as counts and percentages. Continuous variables were expressed as mean and standard deviations or medians with range. The differences were considered to be statistically significant at *p*-value of <0.05. Chi-squared test and the analysis of variance (ANOVA, between- and within-subject) were performed, if necessary.

Independent Bioethics Committee for Scientific Research at Medical University of Gdańsk approved the study protocol (No. NKBBN/192/2019, 10 April 2019). Declaration of Helsinki was followed. All patients gave their written informed consent.

## 3. Results

### 3.1. Patient Characteristics and Complications

Out of 114 patients with apical prolapse, 1 (0.9%) was diagnosed with POP-Q II stage, 84 (73.7%) with POP-Q III and 29 (25.4%) with POP-Q IV. The mean age and BMI were 62 ± 7.6 years and 27.3 ± 3.9 kg/m^2^ for LP and 62 ± 8.7 years and 27.6 ± 4.1 kg/m^2^ for SSLF, respectively. The mean operative time and hospital stay after surgery were 151.8 ± 36.2 min and 2.6 ± 1.1 days for LP and 69 ± 20.4 min and 2.7 ± 1.0 days for SSLF, respectively. In the LP group, 35 (63.6%) women underwent concurrent supracervical hysterectomy. The mean operative time was statistically shorter for SSLF (*p* < 0.001). Concurrent procedures for other compartments were also performed more often in SSLF patients (*p* < 0.001). The overall complication rate did not differ between the groups (*p* = 0.7). Severe intraoperative complications, defined as ≥III grade C-D, occurred in two (1.8%) patients in the entire study population—one opening of the Douglas pouch in SSLF and one bowel injury in the LP group, both sutured during surgery, without further consequences. The most common adverse event after SSLF was buttock pain in 9 (14.8%) patients and reported within 30 days of hospital discharge. Two cases of severe late-onset complications were observed in the LP group: one hernia in the scar of surgical trocar and one vaginal evisceration. There was no statistically significant difference in hemoglobin change and inpatient stay between the groups. The characteristics of the study participants and perioperative data are presented in Table 1.

### 3.2. Urinary Incontinence

Data on urinary incontinence among the study population are presented in Table 2. No statistically significant differences in the rates of previous UI surgery were found. A concurrent SUI surgery was not performed. At the follow-up visit, no differences between persistent and de novo SUI or UUI (*p* > 0.05) were observed in either group. After apical repair, LP patients were more often treated surgically with transobturator tape (TOT) as a management of persistent symptomatic SUI, as compared to SSLF patients (*p* = 0.01).

### 3.3. Anatomical Results

Significant postoperative improvement was observed for all POP-Q points: Aa, Ba, Ap, Bp, and C in both groups (*p* < 0.03). However, SSLF patients had significantly smaller improvement in Aa point (*p* = 0.003). The shortening of TVL was found in both groups, but predominantly in SSLF patients (Table 3). At follow-up, no statistically significant differences in the improvement of the apical defect between both groups were found (*p* > 0.05).

The mean follow-up was 26.9 ± 12 and 37.3 ± 17.5 months for LP and SSLF, respectively (Table 4). Anatomical success was achieved in 40 (75.5%) LP and 36 (59%) SSLF patients. No significant differences in surgical failure rates between both groups were observed (*p* = 0.06), but reoperations due to apical relapse were more frequent in the SSLF group (*p* = 0.02). Mesh-related complications, such as exposure, occurred in two (3.8%) patients in the LP group.

### 3.4. Subjective Results

The analysis of PGI-I scores revealed that postoperative improvement (*a little better, much better or very much better*) was reported by 40 (75.5%) and 44 (72.1%) patients in the LP and SSLF groups, respectively. No change was reported by 10 (18.9%) LP and 13 (21.3%) SSLF patients, whereas 3 (5.7%) LP and 4 (6.5%) SSLF patients felt worse than at baseline. The overall PGI-I did not differ statistically significantly between both groups. A positive general recommendation (*yes, I would* and *rather yes*) was expressed by 36 (68%) and 40 (65.6%) of LP and SSLF patients, respectively, whereas 1 (1.9%) and 3 (5%) patients in the SSLF and LP groups declared significant dissatisfaction (*very unsatisfied*) with surgery (Table 5).

Pain intensity at follow-up was lower in SSLF patients as compared to LP (*p* = 0.03). Both procedures did not aggravate constipation postoperatively. In addition, 23 (95.8%) and 26 (96.3%) patients in the LP and SSLF groups remained sexually active after surgery (*p* = 0.93).

PFDI-20 and PFIQ-7 total scores improved after surgery (*p* < 0.04) in both groups. All three domains (prolapse, colorectal and urinary) of the PFDI-20 questionnaire also improved significantly in both groups (*p* < 0.02). As far as the prolapse domain was concerned, no significant differences in the improvement of prolapse distress (POPDI) scores (LP, −20.8 ± 22.88; SSLF, −15.4 ± 30.0) were found between the groups, despite within-group improvement. As for PFIQ-7, significant improvement after surgery was observed only for the prolapse domain (POPIQ) in both groups (*p* < 0.01) (Table 6). Importantly, a comparison of the changes in the mean domain scores and the total questionnaire scores revealed no statistically significant differences between the two operative methods (*p* > 0.05). The sensation of vaginal bulge (EPIQ #35) was significantly reduced (*p* < 0.001), without no differences between both groups. The analysis of the ISI results also confirmed no postoperative deterioration of UI symptoms between and within both groups.

## 4. Discussion

Treatment effectiveness is a vital condition to achieve long-term success in POP management. Abdominal or laparoscopic sacrocolpopexy (SCP) or sacrocervicopexy (SCerP), as well as vaginal USLS, SSLF or SSHP are the most common procedures to support the apical compartment. Laparoscopic pectopexy, which has recently attracted the attention of various authors, should be considered as a surgical option for apical prolapse repair [12,13,17,18,20,21,32]. Due to mesh position in LP and suture placement in SSLF, the risk of de novo constipation rate is negligible as compared to SCP or SCerP [12,13,18]. All of the procedures are associated with their own benefits and risks. According to a reference medical center in Germany, where LP has been performed since 2007, safety and effectiveness rates for LP are comparable to SCP [13,32]. In a previous study, the learning curve and surgical complications for LP in daily clinical practice had been precisely estimated and confirmed the applicability of this method [17].

In the absence of prospective, randomized, multicenter trials, the comparison of SSLF and LP poses a considerable challenge. In our observational prospective study among 114 patients, we found no differences between the 2 groups as far as anatomical and subjective success and surgical failure rates were concerned. However, we did observe a significantly higher number of reoperations due to apical relapse after SSLF as compared to LP, which may have resulted from a longer follow-up in the SSLF group. To the best of our knowledge, only one retrospective, observational study comparing SSLF (*n* = 43) with LP (*n* = 36), with a mean follow-up of 17.1 ± 10.3 [7–43] months for SSLF and 13.1 ± 6.4 [7–30] months for LP, has been published to date [18]. In that study, apical relapse was defined as POP-Q ≥ 2 and occurred in six (14%) SSLF and four (11.1%) LP patients. Based on these results, the apical relapse rate for the two procedures did not differ, which is consistent with our findings.

There is no consensus in the definitions of surgical failure and POP recurrence used in the literature. Favre-Inhofer et al. [2] analyzed the results of 59 patients after SSLF at 2 follow-up periods and defined failure as anatomical prolapse POP-Q > 1 or repeated surgery. They showed the apical recurrence rate of 3% at 1–5 years following surgery and found no relapses at 5–10 years of follow-up. The anterior vaginal wall was the most common site of recurrence—9 (26%) and 4 (16%) cases at 1–5 years and 5–10 years of follow-up, respectively. However, the response rate in their study was low, with 61 out of 120 subjects who underwent surgery being lost to follow-up. According to Paraiso et al. [33], who analyzed 243 patients after SSLF, relapse was defined as a symptomatic POP-Q 1 or any POP-Q ≥ 2 prolapse. In their study, apical recurrence was found in 20 (8.2%) patients-9 (3.7%) POP-Q 3 and 5 (2.1%) POP-Q 2, for mean follow-up of 73.6 ± 44.8 [1–190] months. Different criteria were used in the OPTIMAL randomized controlled trial, which defined anatomical relapse as apical descent of ≥ one-third of the TVL or anterior or posterior vaginal wall beyond the hymen or retreatment for prolapse or bothersome bulge symptoms [7]. At 3 and 5 years of follow-up after surgical intervention (USLS: *n* = 188 or SSLF: *n* = 186), the surgical failure rate for SSLF was 60.4% and 70.3%, respectively. The 145 failures based on POP-Q measures involved the apex only (27%), anterior or posterior compartment only (34%) or both apex and anterior/posterior compartments (39%). In their study, Jelovsek et al. emphasized the role of follow-up time in calculating the success rates. Compared with the outcomes at 2 years follow-up, the rates of surgical failure increased during the follow-up period. The estimated median time to failure was 1.8 years and 2.6 years for SSLF and USLS, respectively [7]. In contrast, a high objective success rate was reported in yet another study about SSLF at 1 and 7 years follow-up—94.28% (*n* = 33/35) and 96% (*n* = 49/51), respectively [34]. Likewise, Colombo et al. [35], presented long-term SSLF results, with mean follow-up of 6.9 ± 1.7 years [range 4–9], and found recurrent apical prolapse (POP-Q ≥ 2) in 5 (8%) patients at 4 months—5 years follow-up (median 1 year) after SSLF.

Data on surgical failures and recurrence rates after LP are limited. Tahaoglu et al. [36] found no evidence of recurrent prolapse at 6 months follow-up in 22 patients. Likewise, Biler et al. [15] reported no recurrent prolapse or mesh exposure at 6 months follow-up in abdominal SCP (*n* = 44), laparoscopic SCP (*n* = 10) and LP (*n* = 16) for apical prolapse groups. Noe et al. in collaboration with 11 medical institutions [16], reported a total success rate of 96.9% for apical repair, with no mesh exposure or mesh-related complications at 12–18 months follow-up (*n* = 264). In 2 studies about LP with preserved uterus, relapse was found in only 2 (5%) patients at 12 months of follow-up [20], and no cases of relapse were reported by Bagli et al. [21], who analyzed their patients 12 months after delivery. The second reason may be the relative novelty of the procedure, as compared to other methods, which limits the amount of data collected from various centers.

In our study, the overall rate of severe surgical complications was low (1.8%). In the LP group, one (1.9%) superficial small bowel injury during surgery, two (3.8%) mesh exposures and one vaginal evisceration were found. The latter two complications did not meet the time criteria for the Clavien–Dindo classification of perioperative complications, so they were listed separately. The case of transvaginal small bowel evisceration was described elsewhere [37]. Astepe et al. [18] reported one patient with ureteral kinking in SSLF (2.3%) and one patient with bladder injury in the LP group (2.8%), amounting to 2.5% of severe complications. These authors noted one (2.8%) mesh exposure at the vaginal apex in the LP group—a patient who had undergone vaginal hysterectomy two years earlier and SCP one year earlier [18]. Biler et al. [15] reported 1 (3.6%) case of hemorrhage during LP (*n* = 16), which did not require blood transfusion. In their prospective, multicenter study, Noe et al. [32] found five (1%) patients with severe complications—one hemorrhage and four cases of organ damage (three bladder lesions and one ureter injury). Tahaoglu et al. [36], in a group of 22 patients who underwent LP, reported 1 (4.5%) conversion to laparotomy due to adhesions and bleeding. No cases of surgical complications were reported by Bagli et al. [21], while rare cases, i.e., intraoperative hemorrhage from corona mortis stopped by bipolar cautery in one (2.7%) patient, were reported by Salman et al. [20] for LP with uterus preservation. As for SSLF-related complications, the literature offers a higher number of reports. In our study, buttock pain was reported by 9 (14.8%) patients within 30 days of discharge from the hospital. At follow-up visits, three (5%) patients reported persistent buttock pain. In the OPTIMAL study, buttock pain was found in 12.4% of the patients immediately after the procedure and 4.3% at 4–6 weeks follow-up [7]. Apart from one (1.6%) patient with opening of the Douglas pouch, no severe complications during surgery were reported for SSLF in our study. Paraiso et al. [33], reported short-term postoperative complications after SSLF in 89 of 243 women recruited for the study (36.6%). Urinary retention, which occurred in 30 (12.4%) patients, was the most common adverse event. Severe complications (1.2%) included pulmonary embolism (0.4%), conversion to laparotomy (0.4%) and readmission due to hematoma (0.4%). Intraoperative bladder or bowel injuries occurred in five studies about SSLF: in one (0.9%) [38], three (1.9%) [39] and nine (0.7%) [40] patients. In a study by Greisen et al. [41], where SSLF was performed as the so-called “fast track” procedure, no serious surgical problems, visceral injury or perioperative blood transfusions were reported. In addition, in a meta-analysis comparing SSLF with sacrocolpopexy, SSLF proved to be associated with a lower complication rate [42].

The anatomical and subjective outcomes after POP repair have received a similar amount of attention in the literature. However, based on the findings of the new investigating tools such as validated questionnaires, it seems safe to hypothesize that patient goals and subjective outcomes may in fact play a more important role in the quality of patient life and their well-being after surgery. Our study showed a lower rate of pain complaints in both groups at follow-up. According to the PGI-I assessment, 75.5% of LP and 72.1% of SSLF patients reported improvement at follow-up, which is consistent with the findings of a recent study from Denmark, where 76% of the patients reported improvement (little, much or very much better) on the PGI-I scale at 6 months after SSLF [41]. Astepe et al. [18] found higher patient satisfaction rates—93% and 91.7% in SSLF and LP, respectively—but their methodology was specified only to some extent. In the OPTIMAL clinical trial comparing SSLF with USLS, the authors found no statistically significant differences in the PGI-I scores between the groups [7]. In our study, statistically significant improvement was observed in all domains of the PFDI-20 questionnaire and in the POPIQ domain of the PFIQ-7 questionnaire for both groups (SSLF and LP). Similarly, in the previously cited studies, there was an improvement in the PFDI-20 questionnaire scores after surgery for SSLF [2,7]. The literature offers a range of questionnaires, rendering it difficult to compare the results [20,37,43]. In two studies on LP, the Prolapse Quality of Life (P-QOL) questionnaire was applied. It confirmed subjective success with significantly improved QoL at 3 and 6 months after surgery [37,43].

SSLF and LP can be combined with anterior and/or posterior colporrhaphy and SUI procedures, if indicated. In our study, concurrent repairs in other compartments were performed significantly more often in the SSLF group. For LP, lack of simultaneous repairs of other compartments might be considered a study limitation. However, that fact allowed us to observe that LP might additionally have a protective effect on the anterior compartment [13,18] as compared to SSLF, which was confirmed in our study for point Aa. Moreover, in a computer simulation, during the Valsalva maneuver, bilateral fixation in LP allows for better physiological positioning of the vaginal cuff and bladder than unilateral SCP [44]. There is no consensus as to whether bilateral SSLF has an advantage over unilateral SSLF in terms of effectiveness and patient subjective outcome. Mehmet Baki et al. [45] demonstrated that bilateral SSLF with mesh allowed to establish adequate pelvic support for genital prolapse and suggested that the vaginal axis may be closer to the original anatomical position. In contrast, Salman et al. [46] proved that unilateral and bilateral SSLF techniques are associated with similar clinical outcomes. In a recently published meta-analysis involving 4120 patients after SSLF and SCP, SSLF was associated with lower success rate (88.32% vs. 91.45%), higher recurrence (11.58% vs. 8.32%) and dyspareunia (14.36% vs. 4.67%) rates, shorter operative time, lower hemorrhage and wound infection rate and lower and fewer gastrointestinal complications [42]. According to the guidelines of the Royal College of Obstetricians and Gynecologist (RCOG) about post-hysterectomy vaginal vault prolapse, SSLF is associated with earlier recovery compared to abdominal SCP, but may not be appropriate in women with short TVL and should be carefully considered in women with pre-existing dyspareunia (evidence class A) [47]. In our study, both groups had shortening in TVL postoperatively, with a significantly greater shortening in the SSLF group (−0.5 ± 0.8 vs. −1.3 ± 1.3).

Surgical treatment of POP may affect the mobility of the vesicourethral junction, but it might also unmask previously occult SUI. Baser et al. investigated the risk of de novo SUI in women treated for POP with SSLF, with simultaneous vaginal hysterectomy and anterior-posterior colporrhaphy (*n* = 73, 24 months follow-up), and confirmed that SSLF did not increase the rate of de novo SUI [48]. Biler et al. [15] reported two de novo urgency and one de novo SUI cases after LP, without statistical significance. In a prospective, multicenter study about LP and native tissue repair, 50% of the patients reported urgency before surgery, whereas 86% of this group had no urgency after surgery (*p* < 0.001). De novo urgency was registered in 4.2% of the patients (*p* > 0.05), and only 2 cases (0.8%) of de novo SUI were detected in the entire cohort [16]. In our study, no concurrent SUI procedures were performed, and no significantly increased rates of de novo SUI, UUI and mixed UI after surgery were observed in both groups.

High response rate at the follow-up visit and low discontinuation rate are the definite strengths of our study. To the best of our knowledge, this study has been the first report to include such a range of validated questionnaires for patient subjective assessment after SSLF and LP. Statistically significantly different follow-up periods between the groups might constitute a limitation to our study. The observational period was significantly longer for the SSLF, as compared to the LP group due to the novelty of the latter procedure. LP was first introduced at our center in 2015. Additionally, regularity of follow-up visits was sometimes compromised due to the locally introduced health measures during the SARS-CoV-2 pandemic.

## 5. Conclusions

SSLF and LP are both applicable in the treatment of apical prolapse, with good anatomical and subjective outcomes and low rates of severe surgical complications. LP might additionally exert a protective effect on the anterior compartment, as compared to SSLF, which had been confirmed in our study for point Aa.

## Figures and Tables

**Table 1:** Patient characteristics and perioperative data.

Variables	LP (*n* = 53)	SSLF (*n* = 61)	*p*-Value
Age (years)	62.3 ± 8.8	61.8 ± 8.7	0.75 ^a^
BMI (kg/m^2^)	27.3 ± 4.0	27.6 ± 4.1	0.75 ^a^
Postmenopausal	46 (86.8%)	55 (90.2%)	0.57 ^b^
Parity	2.3 ± 1.1	2.5 ± 1.1	0.2 ^a^
	2 [0–7]	2 [1–7]	
Pre-operative POP-Q stage			
2	1 (1.9%)	0	0.34 ^b^
3	41 (77.3%)	43 (70.5%)	
4	11 (20.8%)	18 (29.5%)	
Previous POP surgery			
anterior colporrhaphy	7 (13.2%)	10 (16.4%)	0.63 ^b^
posterior colporrhaphy	6 (11.3%)	12 (19.7%)	0.22 ^b^
anterior vaginal repair with mesh	0	1 (1.6%)	0.35 ^b^
posterior vaginal repair with mesh	1 (1.9%)	0	0.28 ^b^
Concurrent POP surgery			**<0.001 ^b^**
anterior colporrhaphy	0	21 (34.2%)	
posterior colporrhaphy	2 (3.8%)	37 (60.7%)	
laparoscopic anterior repair	2 (3.8%)	X	
laparoscopic posterior repair	1 (1.9%)	X	
Operative time (min)	151.8 ± 36.2	69 ± 20.4	**<0.001 ^a^**
Change in hemoglobin level (g/dL)	1.5 ± 0.5	1.5 ± 0.6	0.76 ^a^
Inpatient stay (days)	2.6 ± 1.1	2.7 ± 1.0	0.42 ^a^
	2 [1–7]	2 [2–6]	
Patients with perioperative complications [C-D]			0.70 ^b^
I	8 (15.1%)	12 (19.7%)	
II	0	0	
III	1 (1.9%)	1 (1.6%)	
IV	0	0	
Total	9 (17%)	13 (21.3%)	

Data presented as mean ± standard deviation, median [range] or *n* (%). Abbreviations: BMI—body mass index; C-D—Clavien–Dindo classification; LP—laparoscopic pectopexy; POP—pelvic organ prolapse; POP-Q—Pelvic Organ Prolapse Quantification; SSLF—sacrospinous ligament suspension; ^a^ between-subject ANOVA test, ^b^ chi-squared test.

**Table 2:** Urinary incontinence data.

Variables	LP (*n* = 53)	SSLF (*n* = 61)	*p*-Value
Previous UI surgery	4 (7.5%)	3 (4.9%)	0.56 ^a^
Transobturator tape	1 (1.9%)	3 (4.9%)	0.38 ^a^
Kelly plication	3 (5.7%)	0	0.06 ^a^
Concurrent SUI surgery	0	0	*-*
UI postoperatively			
SUI de novo	5 (9.4%)	5 (8.2%)	0.82 ^a^
SUI persistent	7 (13.2%)	5 (8.2%)	0.38 ^a^
UUI de novo	0	2 (3.3%)	0.18 ^a^
UUI persistent	5 (9.4%)	4 (6.5%)	0.57 ^a^
Mixed UI de novo	2 (3.8%)	2 (3.3%)	0.88 ^a^
Mixed UI persistent	9 (17%)	12 (19.7%)	0.71 ^a^
Positive cough stress test			
de novo	2 (3.8%)	5 (8.2%)	0.33 ^a^
persistent	10 (18.9%)	15 (24.6%)	0.46 ^a^
Rehospitalizations with MUS insertion			
total	5 (9.4%)	1 (1.6%)	0.06 ^a^
transobturator tape	5 (9.4%)	0	**0.01 ^a^**
retropubic tape	0	1 (1.6%)	0.35 ^a^

Data presented as *n* (%). Abbreviations: UI—urinary incontinence; LP—laparoscopic pectopexy; MUS—midurethral sling; SSLF—sacrospinous ligament suspension; SUI—stress urinary incontinence; UUI—urgency urinary incontinence; ^a^ chi-squared test.

**Table 3:** POP-Q measurements: baseline and at follow-up (in centimeters).

	LP (*n* = 53)				SSLF (*n* = 61)				Comparison of Change between Groups
	Pre	Post	Change	*p*-Value	Pre	Post	Change	*p*-Value	*p*-Value
Aa	1.7 ± 2.1	−0.4 ± 2.6	−2.1 ± 2.4	**<0.001 ^a^**	1.0 ± 2.0	−0.2 ± 2.3	−0.8 ± 2.3	**0.01 ^a^**	**0.003 ^b^**
Ba	3.6 ± 4.0	0.7 ± 4.0	−3.0 ± 4.0	**<0.001 ^a^**	3.7 ± 3.4	1.4 ± 3.5	−2.3 ± 3.6	**<0.001 ^a^**	0.37 ^b^
Ap	0.1 ± 2.2	−1.2 ± 1.9	−0.8 ± 2.6	**0.03 ^a^**	0.02 ± 2.3	−2.2 ± 1.2	−1.5 ± 2.8	**<0.001 ^a^**	0.17 ^b^
Bp	2 ± 4.5	−0.6 ± 3.1	−2.1 ± 4.4	**<0.001 ^a^**	2.1 ± 4.1	−1.7 ± 2.3	−3.0 ± 4.6	**<0.001 ^a^**	0.25 ^b^
C	5.4 ± 3.0	−4.2 ± 4.6	−8.6 ± 5.1	**<0.001 ^a^**	5.6 ± 2.6	−3.4 ± 4.8	−3.7 ± 3.4	**<0.001 ^a^**	0.09 ^b^
TVL	9.5 ± 1.4	9.0 ± 1.5	−0.5 ± 0.8	**<0.001 ^a^**	9.8 ± 1.6	8.8 ± 1.6	−1.3 ± 1.3	**<0.001 ^a^**	**0.001 ^b^**

Data presented as mean ± standard deviation. Abbreviations: LP—laparoscopic pectopexy; SSLF—sacrospinous ligament suspension; ^a^ within-subject ANOVA test, ^b^ between-subject ANOVA test.

**Table 4:** Assessment of the surgical effects, reoperations and mesh complications.

Variables	LP (*n* = 53)	SSLF (*n* = 61)	*p*-Value
Follow-up (months)	26.9 ± 12	37.3 ± 17.5	**<0.001 ^a^**
	25 [5–50]	39 [6–99]	
Surgical failure POP-Q C ≥ −1	13 (24.5%)	25 (41%)	0.06 ^b^
Reoperation for apical prolapse			
Bilateral SSHP with graft	2 (3.8%)	1 (1.6%)	
LP	3 (5.7%)	11 (18%)	
SSLF	0	3 (5%)	
LeFort colpocleisis	0	1 (1.6%)	
Total	5 (9.4%)	16 (26.2%)	**0.02 ^b^**
Mesh complications		X	-
mesh detachment	7 (13.2%)		
mesh exposure	2 (3.8%)		

Data presented as mean ±standard deviation, median [range] or *n* (%). Abbreviations: LP—laparoscopic pectopexy; SSLF—sacrospinous ligament suspension; ^a^ between-subject ANOVA test, ^b^ chi-squared test.

**Table 5:** Subjective outcomes—part 1.

Variables	LP (*n* = 53)	SSLF (*n* = 61)	*p*-Value
Change in pain intensity (VAS, score 0–10)	0.03 ± 1.8	−0.67 ± 1.5	**0.03 ^a^**
Constipation			
de novo	1 (1.9%)	2 (3.3%)	0.64 ^b^
persistent	16 (30.2%)	17 (27.9%)	0.78 ^b^
Sexually active			
preoperative	24 (45.3%)	27 (44.3%)	0.91 ^b^
postoperative	23 (43.4%)	26 (42.6%)	0.93 ^b^
Satisfaction with surgery			0.26 ^b^
very satisfied	21 (39.6%)	16 (26.2%)	
satisfied	11 (20.7%)	22 (36.1%)	
neutral	6 (11.3%)	8 (13.1%)	
unsatisfied	14 (26.4%)	12 (20%)	
very unsatisfied	1 (1.9%)	3 (5%)	
Willingness to suggest such surgery to a friend with a similar problem			0.98 ^b^
yes, I would	31 (58.5%)	34 (55.7%)	
rather yes	5 (9.4%)	6 (10%)	
I am not sure	5 (9.4%)	5 (8.2%)	
rather not	3 (5.7%)	3 (5%)	
no	9 (17%)	13 (21.3%)	
PGI-I			0.08 ^b^
very much better	21 (39.6%)	20 (32.8%)	
much better	7 (13.2%)	16 (26.2%)	
a little better	12 (22.6%)	8 (13.1%)	
same as before surgery	10 (18.9%)	13 (21.3%)	
a bit worse	0	4 (6.5%)	
much worse	3 (5.7%)	0	
very much worse	0	0	

Data presented as mean ± standard deviation or *n* (%). Abbreviations: LP—laparoscopic pectopexy; PGI-I—Patient Global Impression of Improvement questionnaire for urogenital prolapse; SSLF—sacrospinous ligament suspension; VAS—visual analogue scale; ^a^ between-subject ANOVA test, ^b^ chi-squared test.

**Table 6:** Subjective outcomes—part 2: mean scores of questionnaires.

	LP (*n* = 53)				SSLF (*n* = 61)				Comparison of Change between Groups
	Pre	Post	Change	*p*-Value	Pre	Post	Change	*p*-Value	*p*-Value
**ISI**	2.0 ± 2.0	1.6 ± 2.1	−0.5 ± 2.1	0.11 ^b^	1.4 ± 1.9	1.5 ± 2.1	0.1 ± 2.0	0.8 ^b^	0.18 ^a^
**EPIQ#35**	8.8 ± 1.9	5.5 ± 3.3	−3.8 ± 3.3	**<0.001 ^b^**	8.4 ± 2.2	5.6 ± 3.2	−2.8 ± 3.2	**<0.001 ^b^**	0.26 ^a^
**PFDI-20**									
POPDI-6	51.7 ± 22.6	20.8 ± 22.5	−20.8 ± 22.8	**<0.001 ^b^**	52.5 ± 21.3	21.1 ± 24.5	−15.4 ± 30.0	**<0.001 ^b^**	0.28 ^a^
CRADI-8	22.2 ± 19.9	16.5 ± 18.8	−5.8 ± 16.9	**0.02 ^b^**	23.1 ± 21.3	17.3 ± 2.0	−5.8 ± 22.0	**0.04 ^b^**	0.99 ^a^
UDI-6	46.5 ± 26.0	25.6 ± 25.2	−31.0 ± 21.0	**<0.001 ^b^**	40.2 ± 27.0	24.9 ± 25.5	−31.3 ± 28.5	**<0.001 ^b^**	0.92 ^a^
Total score	120.3 ± 60.0	62.8 ± 57.3	−57.5 ± 51.4	**<0.001 ^b^**	115.7 ± 54.7	63.2 ± 56.0	−52.5 ± 64.8	**<0.001 ^b^**	0.65 ^a^
**PFIQ-7**									
UIQ-7	27.8 ± 29.5	21.2 ± 28.2	−6.6 ± 32.5	0.14 ^b^	26.5 ± 30.0	19.5 ± 28.2	−7.0 ± 32.1	0.09 ^b^	0.95 ^a^
CRAIQ-7	11.6 ± 23.4	11.3 ± 20.6	−9.6 ± 31.8	0.09 ^b^	13.0 ± 20.5	9.0 ± 17.4	−6.6 ± 31.4	0.15 ^b^	0.61 ^a^
POPIQ-7	31.6 ± 24.0	16.1 ± 23.5	−10.4 ± 33.1	**<0.001 ^b^**	37.5 ± 25.7	14.4 ± 23.3	−18.0 ± 35.3	**<0.001 ^b^**	0.24 ^a^
Total score	71.0 ± 68.1	48.6 ± 64.2	−7.4 ± 90.1	**0.04 ^b^**	77.0 ± 64.2	43.0 ± 54.3	−18.5 ± 89.4	**<0.001 ^b^**	0.51 ^a^

Data presented as mean ± standard deviation. Abbreviations: CRADI-8—Colorectal-Anal Distress Inventory; CRAIQ-7—Colo-Rectal-Anal Impact Questionnaire; EPIQ #35—Epidemiology of Prolapse and Incontinence Questionnaire; ISI—Incontinence Severity Index; LP—laparoscopic pectopexy; PFDI-20—Pelvic Floor Distress Inventory questionnaire; PFIQ-7—Pelvic Floor Impact Questionnaire; POPDI-6—Pelvic Organ Prolapse Distress Inventory; POPIQ-7—Pelvic Organ Prolapse Impact Questionnaire; UDI-6—Urogenital Distress Inventory; UIQ-7—Urinary Impact Questionnaire; SSLF—sacrospinous ligament suspension; ^a^ between-subject ANOVA test, ^b^ within-subject ANOVA test.

## Data Availability

The data presented in this study are available on request from the corresponding author. The data are not publicly available due to ongoing study.

## References

[1] Mattsson N.K., Karjalainen P.K., Tolppanen A.M., Heikkinen A.M., Sintonen H., Härkki P., Nieminen K., Jalkanen J. (2020). Pelvic organ prolapse surgery and quality of life-a nationwide cohort study. Am. J. Obstet. Gynecol..

[2] Favre-Inhofer A., Carbonnel M., Murtada R., Revaux A., Asmar J., Ayoubi J.-M. (2021). Sacrospinous ligament fixation: Medium and long-term anatomical results, functional and quality of life results. BMC Women’s Health.

[3] Paz-Levy D., Yohay D., Neymeyer J., Hizkiyahu R., Weintraub A.Y. (2017). Native tissue repair for central compartment prolapse: A narrative review. Int. Urogynecol. J..

[4] Dällenbach P., Kaelin-Gambirasio I., Jacob S., Dubuisson J.B., Boulvain M. (2008). Incidence rate and risk factors for vaginal vault prolapse repair after hysterectomy. Int. Urogynecol. J. Pelvic Floor Dysfunct..

[5] Aboseif C., Liu P. (2021). Pelvic Organ Prolapse. StatPearls.

[6] Karjalainen P.K., Mattsson N.K., Nieminen K., Tolppanen A.M., Jalkanen J.T. (2019). The relationship of defecation symptoms and posterior vaginal wall prolapse in women undergoing pelvic organ prolapse surgery. Am. J. Obstet. Gynecol..

[7] Jelovsek J.E., Barber M.D., Brubaker L., Norton P., Gantz M., Richter H.E., Weidner A., Menefee S., Schaffer J., Pugh N. (2018). NICHD Pelvic Floor Disorders Network. Effect of Uterosacral Ligament Suspension vs Sacrospinous Ligament Fixation with or without Perioperative Behavioral Therapy for Pelvic Organ Vaginal Prolapse on Surgical Outcomes and Prolapse Symptoms at 5 Years in the OPTIMAL Randomized Clinical Trial. JAMA.

[8] Hamdy M.A., Ahmed W.A.S., Taha O.T., Abolill Z.M., Elshahat A.M., Aboelroose A.A. (2019). Late suture site complications of sacrospinous ligament fixation. Eur. J. Obstet. Gynecol. Reprod. Biol..

[9] Tseng L.H., Chen I., Chang S.H., Lee C.-L. (2013). Modern role of sacrospinous ligament fixation for pelvic organ prolapse surgery—A systemic review. Taiwan J. Obstet. Gynecol..

[10] Richter K. (1967). The surgical treatment of the prolapsed vaginal fundus after uterine extirpation. A contribution on Amreich’s the sacrotuberal vaginal fixation. Die operative Behandlung des prolabierten Scheidengrundes nach Uterusexstirpation. Ein Beitrag zur Vaginaefixatio sacrotuberalis nach Amreich. Geburtshilfe Frauenheilkd.

[11] Joshi V.M. (1993). A new technique of uterine suspension to pectineal ligaments in the management of uterovaginal prolapse. Obstet. Gynecol..

[12] Banerjee C., Noé K.G. (2011). Laparoscopic pectopexy: A new technique of prolapse surgery for obese patients. Arch. Gynecol. Obstet..

[13] Noé K.G., Spüntrup C., Anapolski M. (2013). Laparoscopic pectopexy: A randomised comparative clinical trial of standard laparoscopic sacral colpo-cervicopexy to the new laparoscopic pectopexy. Short-term postoperative results. Arch. Gynecol. Obstet..

[14] Noé K.G., Schiermeier S., Alkatout I., Anapolski M. (2015). Laparoscopic pectopexy: A prospective, randomized, comparative clinical trial of standard laparoscopic sacral colpocervicopexy with the new laparoscopic pectopexy-postoperative results and intermediate-term follow-up in a pilot study. J. Endourol..

[15] Biler A., Ertas I.E., Tosun G., Hortu I., Turkay U., Gultekin O.E., Igci G. (2018). Perioperative complications and short-term outcomes of abdominal sacrocolpopexy, laparoscopic sacrocolpopexy, and laparoscopic pectopexy for apical prolapse. Int. Braz. J. Urol..

[16] Noé G.K., Schiermeier S., Papathemelis T., Fuellers U., Khudyakovd A., Altmann H.H., Borowski S., Morawski P.P., Gantert M., De Vree B. (2021). Prospective International Multicenter Pelvic Floor Study: Short-Term Follow-Up and Clinical Findings for Combined Pectopexy and Native Tissue Repair. J. Clin. Med..

[17] Szymczak P., Grzybowska M.E., Sawicki S., Wydra D.G. (2021). Laparoscopic Pectopexy-CUSUM Learning Curve and Perioperative Complications Analysis. J. Clin. Med..

[18] Astepe B.S., Karsli A., Köleli I., Aksakal O.S., Terzi H., Kale A. (2019). Intermediate-term outcomes of laparoscopic pectopexy and vaginal sacrospinous fixation: A comparative study. Int. Braz. J. Urol..

[19] Developed by the Joint Writing Group of the American Urogynecologic Society and the International Urogynecological Association (2020). Joint report on terminology for surgical procedures to treat pelvic organ prolapse. Int. Urogynecol. J..

[20] Salman S., Kumbasar S., Yeniocak A.S. (2022). Uterine preserving technique in the treatment of pelvic organ prolapse: Laparoscopic pectopexy. J. Obstet. Gynaecol. Res..

[21] Bagli I., Tahaoglu E.A. (2020). Pregnancy outcomes after laparoscopic pectopexy surgery: A case series. J. Obstet. Gynaecol. Res..

[22] Lykke R., Blaakær J., Ottesen B., Gimbel H. (2015). The indication for hysterectomy as a risk factor for subsequent pelvic organ prolapse repair. Int. Urogynecol. J..

[23] https://www.bsge.org.uk/news/nice-consultation-document-mesh-pectopexy/.

[24] Haylen B.T., Maher C.F., Barber M.D., Camargo S., Dandolu V., Digesu A., Goldman H.B., Huser M., Milani A.L., Moran P.A. (2016). An International Urogynecological Association (IUGA)/International Continence Society (ICS) joint report on the terminology for female pelvic organ prolapse (POP). Int. Urogynecol. J..

[25] Clavien P.A., Barkun J., de Oliveira M.L., Vauthey J.N., Dindo D., Schulick R.D., Santibanes E., Pekolj J., Slankamenac K., Bassix C. (2009). The Clavien-Dindo classification of surgical complications: Five-year experience. Ann. Surg..

[26] Grzybowska M.E., Rechberger T., Wrobel A., Baranowski W., Stangel-Wojcikiewicz K., Rogowski A., Kluz T., Narojczyk-Swiesciak E., Wlazlak E., Burzynski B. (2021). The Urogynecology Section of the Polish Society of Gynecologists and Obstetricians guidelines on the management of non-neurogenic overactive bladder syndrome in women. Ginekol. Pol..

[27] Srikrishna S., Robinson D., Cardozo L. (2010). Validation of the patient global impression of improvement (PGI-I) for urogenital prolapse. Int. Urogynecol. J..

[28] Sandvik H., Seim A., Vanvik A., Hunskaar S. (2000). A severity index for epidemiological surveys of female urinary incontinence: Comparison with 48-hour pad-weighing tests. Neurourol. Urodyn..

[29] Bochenska K., Grzybowska M.E., Piaskowska-Cala J., Mueller M., Lewicky-Gaupp C., Wydra D., Kenton K. (2021). Translation and validation of the Polish version of the Pelvic Floor Impact Questionnaire short form 7. Int. Urogynecol. J..

[30] Grzybowska M.E., Griffith J.W., Kenton K., Mueller M., Piaskowska-Cala J., Lewicky-Gaupp C., Wydra D., Bochenska K. (2019). Validation of the Polish version of the Pelvic Floor Distress Inventory. Int. Urogynecol. J..

[31] Grzybowska M.E., Piaskowska-Cala J., Wydra D.G. (2019). Polish translation and validation of the Pelvic Organ Prolapse/Urinary Incontinence Sexual Questionnaire, IUGA-Revised (PISQ-IR). Int. Urogynecol. J..

[32] Noé G.K., Schiermeier S., Papathemelis T., Fuellers U., Khudyakov A., Altmann H.H., Borowski S., Morawski P.P., Gantert M., De Vree B. (2020). Prospective international multicenter pectopexy trial: Interim results and findings post surgery. Eur. J. Obstet. Gynecol. Reprod. Biol..

[33] Paraiso M.F., Ballard L.A., Walters M.D., Lee J.C., Mitchinson A.R. (1996). Pelvic support defects and visceral and sexual function in women treated with sacrospinous ligament suspension and pelvic reconstruction. Am. J. Obstet. Gynecol..

[34] Aksakal O., Doğanay M., Onur Topçu H., Kokanali K., Erkilinç S., Cavkaytar S. (2014). Long-term surgical outcomes of vaginal sacrospinous ligament fixation in women with pelvic organ prolapse. Minerva Chir..

[35] Colombo M., Milani R. (1998). Sacrospinous ligament fixation and modified McCall culdoplasty during vaginal hysterectomy for advanced uterovaginal prolapse. Am. J. Obstet. Gynecol..

[36] Tahaoglu A.E., Bakir M.S., Peker N., Bagli İ., Tayyar A.T. (2018). Modified laparoscopic pectopexy: Short-term follow-up and its effects on sexual function and quality of life. Int. Urogynecol. J..

[37] Szymczak P., Wydra D.G. (2021). Evisceration of the small intestine through the vagina as a rare complication after laparoscopic pectopexy. Ginekol. Pol..

[38] Hefni M., El-Toukhy T., Bhaumik J., Katsimanis E. (2003). Sacrospinous cervicocolpopexy with uterine conservation for uterovaginal prolapse in elderly women: An evolving concept. Am. J. Obstet. Gynecol..

[39] Pasley W.W. (1995). Sacrospinous suspension: A local practitioner’s experience. Am. J. Obstet. Gynecol..

[40] Sze E.H., Karram M.M. (1997). Transvaginal repair of vault prolapse: A review. Obstet. Gynecol..

[41] Greisen S., Axelsen S.M., Bek K.M., Guldberg R., Glavind-Kristensen M. (2021). Fast track sacrospinous ligament fixation: Subjective and objective outcomes at 6 months. BMC Women’s Health.

[42] Zhang W., Cheon W.C., Zhang L., Wang X., Wei Y., Lyu C. (2022). Comparison of the effectiveness of sacrospinous ligament fixation and sacrocolpopexy: A meta-analysis. Int. Urogynecol. J..

[43] Karslı A., Karslı O., Kale A. (2021). Laparoscopic Pectopexy: An Effective Procedure for Pelvic Organ Prolapse with an Evident Improvement on Quality of Life. Prague Med. Rep..

[44] Bhattarai A., Staat M. (2020). A computational study of organ relocation after laparoscopic pectopexy to repair posthysterectomy vaginal vault prolapse. Comput. Methods Biomech. Biomed. Eng. Imaging Vis..

[45] Baki M., Hakan Güraslan S., Çakmak Y., Ekin M. (2015). Bilateral sacrospinous fixation without hysterectomy: 18-month follow-up. J. Turk. Ger. Gynecol. Assoc..

[46] Salman S., Babaoglu B., Kumbasar S., Bestel M., Ketenci Gencer F., Tuna G., Besimoglu B., Yüksel S., Uçar E. (2019). Comparison of Unilateral and Bilateral Sacrospinous Ligament Fixation Using Minimally Invasive Anchorage. Geburtshilfe Frauenheilkd.

[47] https://www.rcog.org.uk/globalassets/documents/guidelines/gtg-46.pdf.

[48] Başer E., Seçkin K.D., Kadiroğullari P., Kiyak H. (2020). The effect of sacrospinous ligament fixation during vaginal hysterectomy on postoperative de novo stress incontinence occurrence: A prospective study with 2-year follow-up. Turk. J. Med. Sci..

